# Single point mutations reveal amino acid residues important for *Chromobacterium violaceum* transaminase activity in the production of unnatural amino acids

**DOI:** 10.1038/s41598-018-35688-7

**Published:** 2018-11-26

**Authors:** Sarah A. Almahboub, Tanja Narancic, Darren Fayne, Kevin E. O’Connor

**Affiliations:** 1 UCD Earth Institute and School of Biomolecular and Biomedical Science, University College Dublin, Belfield, Dublin 4 Ireland; 2 BEACON - Bioeconomy Research Centre, Ireland, University College Dublin, Belfield, Dublin 4 Ireland; 3 Molecular Design Group, School of Biochemistry and Immunology, Trinity Biomedical Sciences Institute, Trinity College Dublin, Dublin 2, Ireland

## Abstract

Unnatural amino acids (UAAs) are chiral amines with high application potential in drug discovery and synthesis of other valuable chemicals. Biocatalysis offers the possibility to synthesise novel optically pure UAAs with different physical and chemical properties. While the biocatalytic potential of transaminases in the synthesis of UAAs has been demonstrated, there is still a need to improve the activity with non-native substrates and to understand which amino acids residues are important for activity with these UAAs. Using a rational design approach, six variants of *Chromobacterium violaceum* DSM30191 transaminase (CV_TA) carrying a single and one variant carrying two substitutions were generated. Among the variants with a single substitution, CV_Y168F showed a 2 to 2.6-fold increased affinity for 2-oxooctanoic acid (2-OOA) and 3-oxobutyric acid (3-OBA) methyl ester used to synthesise an α- and β-UAA. Analysis of the first half of the transaminase reaction showed no change in the activity with the donor (*S*)-1-phenylethylamine. The combination of W60C and Y168F substitutions improved the CV_TA affinity for 2-OOA 10-fold compared to the wild type. Other substitutions showed no change, or reduced activity with the tested substrates. Our findings provide structural information on CV_TA and demonstrate the potential of rational design for biosynthesis of UAAs.

## Introduction

The use of transaminases (TAs) in manufacturing optically pure chiral amines has been identified as one of the key emerging areas for the pharmaceutical industry^1–3^. Unnatural amino acids (UAAs) as chiral amines can be applied in the manufacture of diverse anticancer agents^4–6^, antimicrobials^7^, agrochemicals and other value added products^6,8^. In addition to their prominent role in peptide research and drug discovery, UAAs can be used to investigate the structure and dynamics of proteins, to study protein interactions, or to control the activity of proteins in living cells^9^. Great interest in UAAs has inspired development of numerous methods for their synthesis, including biocatalysis. While the biocatalytic potential of TAs in the synthesis of UAAs has been demonstrated^7,10,11^, there is a growing need to expand the variety of UAAs which could be achieved by engineering existing TAs. While some TAs exhibit a naturally wide substrate range, the activity towards non-native substrates is usually tens of folds lower compared to the native substrate^12^.

TAs catalyse the transfer of the amino group from an amino donor to an amino acceptor to produce a chiral amine by a reductive amination reaction. Pyridoxal-5′-phosphate (PLP) is used as a cofactor and acts as an intermediate amino group acceptor and an electron sink^13^. In all known TAs the active site consists of a large and a small binding pocket^14^. The small binding pocket allows entry of a substituent no larger than an ethyl group^14,15^. However, Kaulmann and co-workers suggested that an ω-TA originating from *Chromobacterium violaceum* DSM30191 (CV_TA) has a wide substrate range with respect to both amino group donors and acceptors^16^.

The crystal structure of CV_TA has been solved^17,18^. CV_TA is an (*S*)-enantioselective enzyme of approximately 100 kDa, that prefers amino group donors with an aromatic ring over aliphatic amines and amino acids^16^. CV_TA shows good activity with both aliphatic and aromatic acceptors, with pyruvate and glyoxylate being the preferred acceptors^16,19,20^. CV_TA is a homodimer in which each monomer has an active site that is formed by amino acid residues of both subunits at the dimeric interface^17,18^. Each active site has a PLP binding pocket and a substrate-binding region. In the holoenzyme form, the cofactor PLP is bound to the CV_TA apoenzyme *via* a Schiff base linkage formed between PLP and the lysine residue K288 in the active site^17^. PLP is further stabilised in the active site by interactions with the aspartic acid D259, which creates a hydrogen bond with the proton on the pyridine ring nitrogen, and pyridine ring itself located between tyrosine Y153 and valine V261. Moreover, a network of hydrogen bonds is formed between the phosphate group of PLP and five active site residues serine S121 and tyrosine Y153 from one monomer, and threonine T321, tyrosine Y322 and asparagine N118 from the other monomer^17^. The substrate-binding pocket contains a large pocket involved in recognition of both hydrophobic and carboxyl groups, and a small pocket to accommodate a side chain of the substrate^14,15,17^.

Due to the availability of the crystal structure of CV_TA and its potential to react with a wide range of substrates this enzyme represents an attractive target for the improvement of the activity with non-native substrates. Consequently, a number of studies have emerged reporting on protein engineering in developing ω-TAs biocatalysts with specific desired characteristics^21–23^.

We have previously shown that CV_TA has activity towards aliphatic 2-oxooctanoic acid (2-OOA) producing 2-aminooctanoic acid which can be used to make anti-bacterial peptides^7^. In the current study, using the reported CV_TA crystal structure^17,18^ and the modelled relationship of CV_TA with 2-OOA as the amino group acceptor, specific amino acid residues were selected as targets for site directed mutagenesis. To test which phase of the reaction was affected, the variants were further analysed by measuring the first half of the transamination reaction i.e. transfer of the amino group to PLP in absence of the keto acceptor. Finally, analysis of the effect of these mutations on the activity of CV_TA with other aliphatic substrates was undertaken to determine if such mutations were specific to 2-OOA or not.

## Results

### *In silico* analysis of the residues important for the CV_TA interaction with 2-oxocotanoic acid (2-OOA)

**Figure 1:**
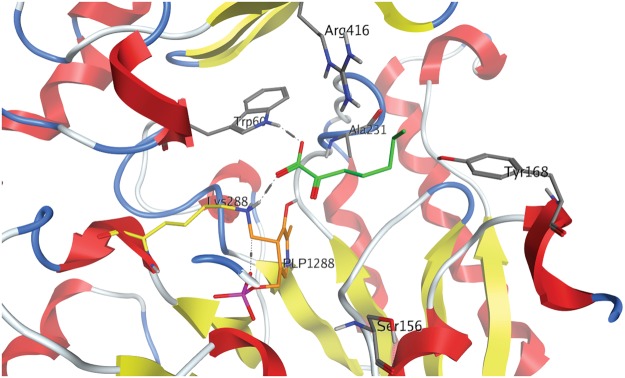
The CV_TA active site and lysine 288 (Lys288 in yellow) residue important for the Schiff’s base formation with PLP (in orange). Residues targeted for site-directed mutagenesis based on the model of CV_TA active site interaction with 2-OOA (green) are displayed in tube rendering and are labelled. The image was generated in MOE using the crystal structure of CV_TA (PDB code: 4AH3).

Using the CV_TA crystal structure^17,18^ and the modelled relationship of CV_TA with 2-OOA as the amino group acceptor, specific amino acid residues were selected as targets for site directed mutagenesis (SDM, Fig. 1).

In order to study the interaction of the active form of CV_TA and 2-OOA we have used the crystal structure of the CV_TA with PLP (PDB: 4AH3^18^). It has been shown that there are significant structure rearrangements between the apo- and holo-enzyme^18^, which led us to use the holo- form of the enzyme with PLP as the enzyme active structure rather than the apoenzyme.

The substrate docking was performed with the dimer after all water molecules were removed, hydrogens were added, and constrained minimisation was applied in order to allow only reasonable bond conformations and afterwards appropriate protonation states of the amino acids and possible hydrogen bonding networks were determined by QuickPrep in MOE 2016.0802 software (Molecular Operating Environment, 2016.0802; Chemical Computing Group Inc.: Montreal, Canada, 2016). 2-OOA was docked into the binding site of CV_TA using MOE (Fig. 1). The protein was rigid but the ligand (2-OOA) was allowed conformational flexibility. The thirty best ranked poses were retained for inspection and the protein ligand interaction fingerprints (PLIFs) were calculated to determine if different interaction patterns could be ascertained.

**Figure 2:**
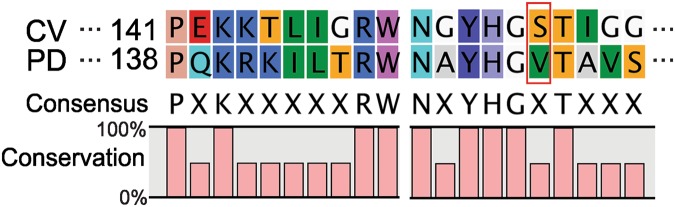
Partial alignment of ω-TA sequence of *C*. *violaceum* (CV; gene: CV_2025) and *P*. *denitrificans* (PD; gene: Pden_3984). Amino acids with similar properties are assigned the same colour based on the CLC sequence viewer 8.0 (www.clcbio.com). The red rectangle designates the residue in CV_TA V153 that corresponds to S156 and that was subjected to SDM.

Based on the docking analysis and visual inspection of the binding orientation of the substrates, two amino acid residues were selected for SDM: tyrosine Y168 and alanine A231 (Fig. 1). The selected residues were then replaced with amino acid residues that would result in either a bigger binding site, or a stabilisation of the acceptor through hydrogen bond formation or creation of a more hydrophobic environment. Arginine R416 seems to be important for coordinating the substrate, and to verify this a conservative substitution to lysine K was made (Fig. 1). Additionally, and based on previous studies, two variants with a single mutation were prepared, CV_W60C and CV_S156A^24,25^. It was previously shown that the substitution of tryptophan at position 60 with cysteine led to the increased specificity of CV_TA for the amino donor 1-PEA^24^. Furthermore, replacing valine at position 153 by alanine in *Paracoccus denitrificans* TA allowed accommodation of linear alkyl substrates^25^. The residue S156 in CV_TA corresponds to V153 in *P*. *denitrificans* that is credited with increasing transaminase enzyme activity (Fig. 2).

### The activity of the CV_TA variants obtained by SDM

Six variants that contained a single amino acid substitution, CV_Y168F, CV_A231S, CV_A231T, CV_S156A, CV_R416K and CV_W60C were generated. To test the effect of amino acid substitutions, the purified variants of CV_TA were analysed in 10 ml biotransformation reactions with 1-PEA as an amino group donor and 2-OOA as the amino acceptor. The reaction rates of the variants were compared with the wild-type enzyme.

#### Enzyme activity of CV_Y168F

**Figure 3:**
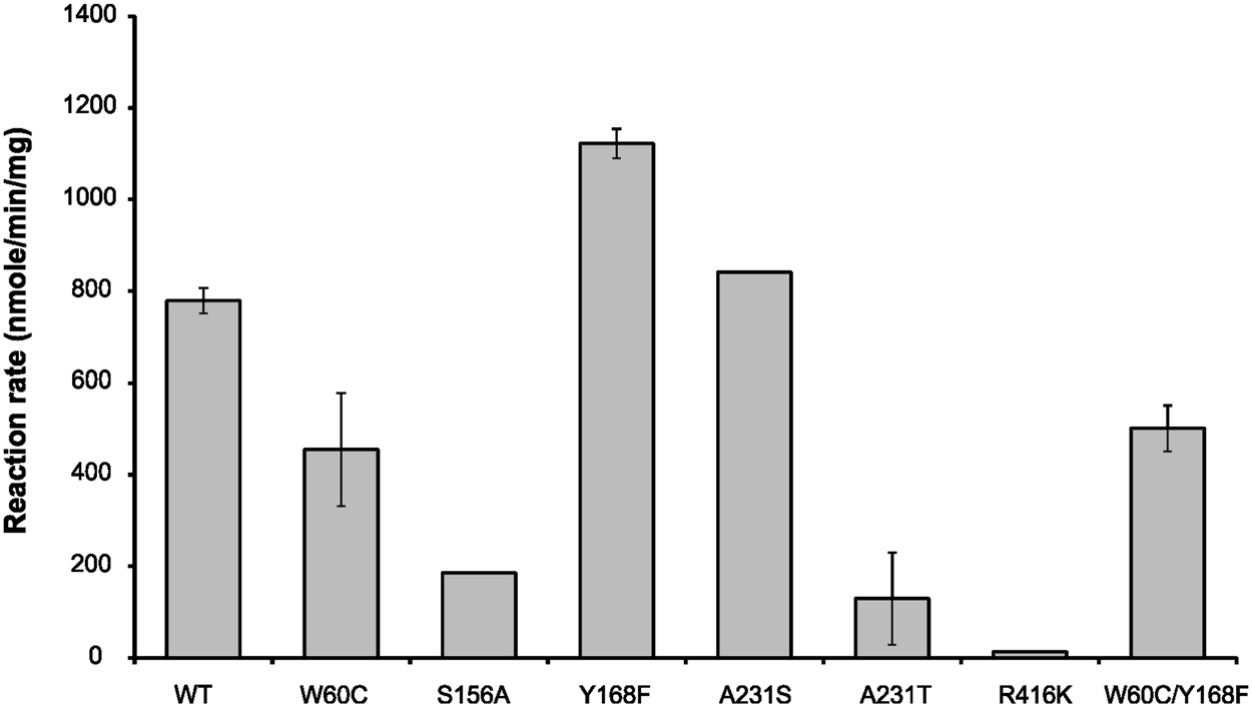
Activity of the CV_TA variants generated by SDM. The reaction rate was monitored by acetophenone assay with 2.5 mM 2-oxooctanoic acid (2-OOA) as the amino acceptor at 45 °C. Data is the average of three independent biological replicates (SD < 5%).

The tyrosine at the position 168 is located opposite to the lysine K288, which forms the Schiff’s base with PLP (Fig. 1). Replacing Y168 with a phenylalanine residue (Y168F) and thus removing the hydroxyl group should allow better interaction of the enzyme and 2-OOA as a hydrophobic substrate (Fig. 1). Indeed, the variant Y168F exhibits 1.5-fold higher activity towards 2-OOA (Fig. 3). It is likely that by replacing tyrosine with phenylalanine creates more space and an increased hydrophobic environment in the active site, which could explain the improved activity towards the 2-OOA.

#### Enzyme activity of CV_A231S and CV_A231T

The docking analysis suggested that replacing the residue alanine 231 with either serine (A231S) or threonine (A231T) as amino acids with polar uncharged side chains would allow stabilisation of the enzyme-substrate interaction through hydrogen bonds. However, variant CV_A231S showed the same activity as wild-type CV_TA, while CV_A231T exhibited 6-fold lower activity when 2-OOA was used as the substrate (Fig. 3).

#### Enzyme activity of CV_S156A

It was previously shown that the mutation V153A in an ω-TA originating from *Paracoccus denitrificans* improved the enzyme activity 4-fold towards 2-OOA^25^. The residue S156 in ω-TA of *C*. *violaceum* is corresponding to the residue V153 in *P*. *denitrificans* (Fig. 2) was substituted with alanine, which resulted in a 4.2-fold decreased activity with 2-OOA.

#### Enzyme activity of CV_R416K

The substitution of R416 with lysine (R416K), also a positively charged but less bulky amino acid led to a 60-fold decrease in the activity (Fig. 2).

#### Enzyme activity of CV_W60C

Tryptophan at position 60 is one of the residues in the small pocket of the substrate-binding region of the CV_TA active site (Fig. 1). It was previously shown that W60 creates a steric barrier for the donors and acceptors^15^ and that replacing W60 with cysteine (W60C) improves the first half of the reaction i.e. deamination of 1-PEA^24^. However, CV_W60C exhibited 1.7-fold lower activity towards 2-OOA compared to the wild-type CV_TA (Fig. 1).

### Kinetic characterisation of the variant CV_Y168F

To investigate the effect of the Y168F substitution on the kinetic characteristics of the transaminase (*K*_*m*_, *K*_*cat*_ and *K*_*cat*_*/K*_*m*_) the purified CV_Y168F was tested using the AP assay and compared with the kinetic parameters of the wild type CV_TA.

CV_Y168F exhibited 2-fold higher affinity and catalytic efficiency towards 2-OOA when compared with the wild-type CV_TA (Table 1).

**Table 1:** Kinetic parameters of purified wild type CV_TA (WT) and CV_Y168F (Y168F) towards the amino group donor 1-PEA and amino acceptor 2-OOA.

Substrate	*K*_*cat*_^c^ (min^−1^)		*K*_*m*_ (mM)		*K*_*cat*_*/K*_*m*_ (min^−1^mM^−1^)	
	WT	Y168F	WT	Y168F	WT	Y168F
1-PEA^a^	28.6 ± 0.3	29.2 ± 5.4	2.6 ± 0.63	1.1 ± 0.3	11.1	25.7
2-OOA^b^	24.7 ± 4.4	21.5 ± 1.7	0.4 ± 0.15	0.2 ± 0.07*	61.8	126.5

^a^Reactions with different 1-PEA concentration: 0.1, 0.25, 0.5, 0.75, 1, 2, 2.5, 4, 10, 15 and 20 mM at 45 °C and pH 7.

^b^Reactions with different amino acceptor concentration: 0.1, 0.25, 0.5, 0.75, 1, 2, 2.5, 4, 10, 15 and 20 mM at 45 °C and pH 7.

^c^*K*_*cat*_ is expressed per active site of CV_TA as it exists as a homodimer with two active sites.

All values are a mean of three independent determinations.

*Statistically significant comparing to the wild type (p < 0.05) by using t-test.

While turnover was similar for the CV_TA and CV_Y168F towards the amino donor 1-PEA, the CV_Y168F variant exhibited 2.4-fold higher affinity (Table 1). This resulted in 2.3-fold increased catalytic efficiency of CV_Y168F variant compared to the wild type enzyme (Table 1).

### Monitoring the first half of the reaction catalysed by variant CV_Y168F

To assess which phase of the reaction was affected by substituting Y168 with F, the kinetic properties of the variant and the wild type CV_TA were assessed using the first half of the TA reaction^24^. In this assay only 1-PEA was used as the substrate and the formation of AP was monitored. The donor was supplied in a range of concentrations from 0.1 to 20 mM, and since there was no acceptor to recycle the cofactor, 250-fold higher concentration of PLP compared to the standard assay was used to allow the detection of AP. As a consequence of no cofactor recycling during the reaction, the molecule of enzyme that performs the half reaction will become inactive. Therefore, a 23-fold higher enzyme concentration, compared to reactions with keto acceptor, was supplied to the reaction compared to standard reaction conditions. The variant CV_W60C was also analysed as it has been shown previously that this substitution resulted in the enzyme having a higher affinity toward 1-PEA when pyruvate was used as amino group acceptor^24^.

The affinity for 1-PEA and catalytic efficiency of the wild-type CV_TA and the variant CV_Y168F were the same in the half reaction (Table 2). This result strongly suggests that the Y168F substitution in the active site improves the second half-reaction with 2-OOA as an acceptor, while not affecting the first-half reaction (Table 2). The substitution W60C improves the first half-reaction as enzyme affinity and turnover number increased 3.3-fold and 4.8-fold respectively, resulting in up to 13-fold increase in catalytic efficiency for the first half of the reaction compared with CV_TA and CV_Y168F.

**Table 2:** Kinetic constants of purified wild type CV_TA (WT), and variants W60C, and Y168F towards 1-PEA* with 5 mM PLP (first half of the TA reaction).

Variant	*K*_cat_** (min^−1^)	*K*_*m*_ (mM)	*K*_*cat*_*/K*_*m*_ (min^−1^mM^−1^)
WT	1.1 ± 0.3	5.7 ± 1.7	0.2
W60C	3.6 ± 0.2	1.2 ± 0.1	2.6
Y168F	1.1 ± 0.1	6 ± 1.6	0.2

*Reactions with different 1-PEA concentration: 0.1, 0.25, 0.5, 0.75, 1, 2, 2.5, 4, 10, 15 and 20 mM, with 1 mg/ml TA at 45 °C and pH 7.

**The *K*_*cat*_ is expressed per active site of CV_TA as it exists as a homodimer with two active sites. All values are a mean of three independent determinations.

### The kinetics of double mutant CV_W60C/Y168F transaminase activity

Given the positive impact of W60C and Y168F substitutions on enzyme activity a CV_TA variant carrying both substitutions was generated. This variant showed 10- and 5-fold increased affinity for 2-OOA compared to the wild type and CV_Y168F (Tables 1 and 3). While the turnover with 2-OOA decreased 2- and 2.3-fold compared to the CV_Y168F and WT, the overall catalytic efficiency of the double mutant was 4.4-fold higher compared to the WT and 2.2-fold higher compared to the single mutant (Tables 1 and 3). CV_W60C/Y168F showed increased catalytic efficiency towards 1-PEA compared with the wild type when half-reaction was monitored, but 3.3-fold lower efficiency compared to the W60C variant (Tables 2 and 3).

**Table 3:** Kinetic parameters of purified CV_W60C/Y168F.

W60C/Y168F	*K*_cat_* (min^−1^)	*K*_*m*_ (mM)	*K*_*cat*_*/K*_*m*_ (min^−1^mM^−1^)
1-PEA^a^	3.4 ± 0.3	4.4 ± 0.1	0.8
2-OOA^b^	10.9 ± 0.7	0.04	272.5

^a^Half-reaction monitored with different 1-PEA concentration: 0.1, 0.25, 0.5, 0.75, 1, 2, 2.5, 4, 10, 15 and 20 mM, with 1 mg/ml TA at 45 °C and pH 7.

^b^Reactions with different 2-OOA concentrations: 0.1, 0.25, 0.5, 0.75, 1, 2, 2.5, 4, 10, 15 and 20 mM at 45 °C and pH 7.

*The *K*_*cat*_ is expressed per active site of CV_TA as it exists as a homodimer with two active sites.

All values are a mean of three independent determinations.

### Enzyme activity of CV_TA variants with a range of aliphatic substrates

We have identified a number of amino acid substitutions that affect CV_TA activity. To understand if these substitutions are specific to 2-OOA as a substrate or if they generally affect enzyme activity a range of aliphatic amino group acceptors were tested with CV_TA and its variants (Fig. 3 and Table S1).

**Figure 4:**
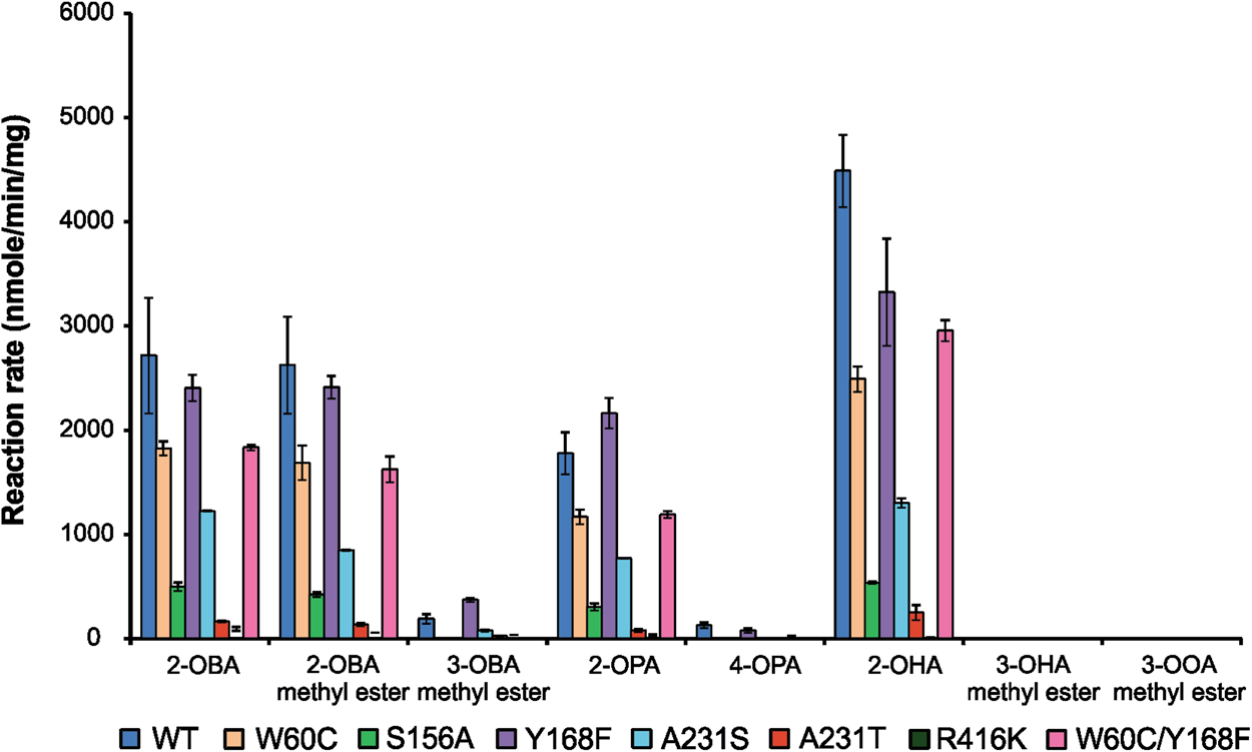
Activity of the CV_TA variants generated by SDM with different aliphatic amino acceptors 2-oxobutyric acid (2-OBA), 2-oxobutyric acid methyl ester (2-OBA methyl ester); 3-oxobutyric acid methyl ester (3-OBA methyl ester); 2-oxopentanoic acid (2-OPA); 4-oxopentanoic acid (4-OPA); 2-oxohexanoic acid (2-OHA); 3-oxohexanoic acid methyl ester (2-OHA methyl ester) and 3-oxooctanoic acid methyl ester (3-OOA methyl ester). The reaction rate was monitored by acetophenone assay with 2.5 mM amino acceptor and 10 mM 1-PEA at 45 °C.

The only improvement in activity of a CV_TA variant was observed in the case of CV_Y168F with 3-oxobutyric acid methyl ester (3-OBA methyl ester), showing 2-fold higher activity compared to the CV_TA (Fig. 4). When tested with other substrates this variant exhibited activity similar to the wild type CV_TA. Other substitutions caused a decrease in activity of corresponding variants. The most profound effect was seen when R416 was replaced with K resulting in a 40 to 260-fold decrease in the activity with aliphatic substrates compared to the wild type (Fig. 4).

**Figure 5:**
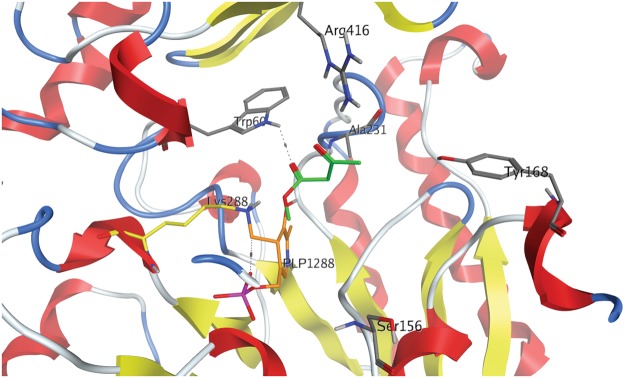
*In silico* modelling of the CV_TA active site and 3-OBA methyl ester as amino acceptor (in green). Lysine 288 (K288 in yellow) residue important for the Schiff’s base formation with PLP (in orange). Residues targeted for site-directed mutagenesis are displayed in tube rendering and are labelled. The image was generated in MOE using the crystal structure of CV_TA (PDB code: 4AH3).

Analysis of the kinetic parameters of CV_Y168F with 3-OBA methyl ester as an amino acceptor revealed that this variant has 2.6-fold higher affinity and 2.4-fold higher catalytic efficiency towards 3-OBA methyl ester compared to the wild type enzyme (Table 4). *In silico* analysis of the CV_TA interaction with 3-OBA methyl ester as amino acceptor suggested that the replacement of Y168 by F could allow better hydrophobic interaction with this substrate (Fig. 5). Interestingly, the double mutant showed no activity with 3-OBA methyl ester (Fig. 4).

**Table 4:** Kinetic parameters of purified CV_ Y168F (Y168F) and CV_TA (WT) with 3-oxobutyric acid methyl ester (3-OBA methyl ester) as amino acceptor.

Substrate	*K*_*cat*_^c^ (min^−1^)^b^		*K*_*m*_ (mM)		*K*_*cat*_*/K*_*m*_ (min^−1^mM^−1^)	
	WT	Y168F	WT	Y168F	WT	Y168F
3-OBA methyl ester^a^	9.3 ± 0.7	8.2 ± 2.6	18.8 ± 5.0	7.1 ± 0.6	0.5	1.2

^a^Reactions with different amino acceptors concentration: 0.1, 0.25, 0.5, 0.75, 1, 2, 2.5, 4, 10, 15 and 20 mM at 45 °C and pH 7.

^b^*K*_*cat*_ is expressed per active site of CV_TA as it exists as a homodimer with two active sites.

Among the α-keto substrates that were tested, CV_TA and variants showed the highest activity towards 2-oxohexanoic acid (2-OHA) followed by 2-oxobutyric acid (2-OBA) and 2-OBA methyl ester (Fig. 4). However, except for CV_Y168F and 3-OBA methyl ester none of the variant-substrate combinations showed improved activity compared to the wild type enzyme (Fig. 4).

The position of the keto group has a major impact on the enzyme activity. While CV_TA and variants showed similar activity with 2-OBA and 2-OBA methyl ester very low activity was observed with 3-OBA methyl ester (Fig. 4). Similarly, no activity was observed with either 3-oxohexanoic acid (3-OHA) methyl ester or 3-oxooctanoic acid (3-OOA) methyl ester, while CV_TA and variants showed activity with corresponding 2-keto acids (Figs 2 and 4). The lack of a commercially available methyl ester of 2-oxohexanoic or 2-oxooctanoic acid did not allow us to further test the structure-function effect of chain length and the presence of a methyl moiety.

## Discussion

The availability of the crystal structure of CV_TA^18^ and the *in silico* analysis performed in this study allowed the rational design of amino acid substitutions which revealed important residues for CV_TA activity. We have targeted 6 amino acid residues of CV_TA which resulted in 6 variants with a single amino acid substitution.

One of the residues located in the active site entrance of CV_TA, tyrosine 168 (Y168) was predicted to potentially cause a hydroxyl group polarity clash with the aliphatic sidechain of 2-OOA. When this residue was replaced with phenylalanine, a variant CV_Y168F was designed and resulted in 2-fold increase in catalytic efficiency with 2-OOA (Table 1). The residue Y168 was found to be highly variable when the CV_TA sequence was compared to 100 ω-TAs sharing >40% identity^23^. However, this residue is part of a second helical turn K167-G173, which together with a loop G152-T157, and a helical turn I158-L163 makes part of the active site^23^. It is hypothesised that changing highly conserved residues would have the most dramatic effect on the activity of an enzyme^23,26^. However, we found that the substitution of the residue Y168, which while part of the active site is not conserved, resulted in a positive effect on the activity of CV_TA (Fig. 1).

It is likely that replacing tyrosine with phenylalanine created more space in the active site along with an increased hydrophobic environment, which potentially allowed better interaction of the enzyme with 2-OOA. However, a resolved crystal structure of the variant CV_Y168F is required to understand the structural rearrangements caused by the tyrosine to phenylalanine substitution and the reason for improved activity. The affinity of the variant CV_Y168F and wild type enzyme for 1-PEA remained the same (Table 2), indicating that the amino acid residue change affected the second half of the reaction which could be explained through stabilisation of the enzyme-acceptor interaction.

The variant CV_W60C, created with the goal of removing a steric barrier formed by tryptophan (W60) positioned in the small binding pocket^27^, was shown to have higher affinity towards 1-PEA (Table 2). However, this change clearly affected only the first half of the reaction, as it gave at least 1.5-fold lower activity with all tested acceptors (Figs 3 and 4). The W60 residue is considered highly conserved among ω-TAs^23^. Replacing W60 with a smaller amino acid cysteine could generate more space in the active site that facilitates the binding of the aromatic amino group acceptor 1-PEA and improves the first half-reaction^27^. It is possible that this increase in catalytic efficiency of the enzyme with the donor is not followed by the increase in the efficiency of the second half-reaction, thus explaining the overall decrease in the reaction efficiency with all tested acceptors.

Combining the W60C substitution with Y168F substitution generated a variant with a higher catalytic efficiency when 2-OOA was used as amino group acceptor (Table 3). However, the same variant showed no activity with 3-OBA methyl ester (Fig. 4) and thus further work is required to explain these differences.

While there are some reports suggesting that the residue arginine R416 is among the conserved key active site residues involved in substrate specificity in ω-TAs^12,15,28–30^, Deszcz and colleagues found that R416 shows a certain degree of variability among ω-TA^23^. It seems to be important for the recognition of the α-carboxylate group of both the amino group donor and acceptor by building a salt bridge and hydrogen bonds^15,18,31^. In the current study, replacing arginine with another positively charged, but less bulky amino acid lysine, generated an enzyme, which retained less than 3% relative activity with all tested substrates, whether they were acids or esters (Figs 3 and 4). The side chain of the R416 residue is positioned inside or outside of the active site depending on the substrate^32^. If the substrate contains a carboxylic group then residue R416 forms a salt bridge with the carboxylate moiety, therefore facilitating deamination by decreasing the energy barrier^32^. If a substrate without a carboxylate moiety is used, the R416 side chain faces outwards^32^. However, this seems to be a specific case with the CV_TA, since when the arginine residue of ω-TA of *V*. *fluvialis* was replaced with lysine the generated variant showed increased activity towards 1-PEA, (*S*)-phenylalanine and (*S*)-aminophenylacetate as amino group donors and pyruvate, oxophenylacetate and 2-oxo-3-phenylpropionate as amino group acceptors^31^.

We speculated that changing the alanine residue A231 into an amino acid with polar uncharged side chains, serine (A231S) or threonine (A231T) would allow stabilisation of the enzyme-substrate interaction through hydrogen bonds. However, no improvement in activity was observed (Fig. 3). This residue when substituted with phenylalanine and in combination with F88A substitution resulted in a substrate-dependent shift in enantioselectivity^27^.

Although the CV_TA and *P*. *denitrificans* share only 38% amino acid identity, their crystal structures are highly similar^33^. However, CV_TA has more space in the active site with higher hydrophobicity that makes the enzyme more active towards aliphatic substrates with medium chain length such as 6-aminohexanoic acid and nonanal^33^. Serine at position 156 in CV_TA is the amino acid which corresponds to the residue V153 in *P*. *denitrificans* (Fig. 2). Nevertheless, designing the substitution which corresponds to V153A resulted in a variant with 4-fold lower activity towards 2-OOA compared to the CV_TA, and lower activity with all tested substrates (Fig. 1).

Based on the *in silico* analysis of the CV_TA–targeted acceptor interactions, amino acid substitutions in this study were selected for their potential to positively impact on transaminase activity. However, only the Y168F substitution proved to be beneficial for activity towards the targeted substrate 2-OOA and 3-OBA methyl ester, while combined W60C/Y168F was only beneficial for 2-OOA as a substrate. The first half of the transaminase reaction is unaffected by the substitution of tyrosine with phenylalanine at residue 168 indicating the improved reaction rate for CV_Y168F is due to the influence on the second half of the reaction. Finally, 2-OOA and 3-OBA methyl ester are interesting substrates as the corresponding amines are an alpha fatty unnatural amino acid and a beta unnatural amino acid respectively with application potential^8^. 2-AOA (2-aminocaprylic acid) is used in the preparation of 1, 5-disubstituted-2-aminoimidazoles, which have antibiotic activity^34^. Furthermore, 2-AOA can be used to design a vaccine delivery system without the need for additional adjuvants^35^ and to modify antimicrobial peptides for the improved activity^7^. 3-aminobutyric acid (3-ABA) or β-homoalanine has been used in the synthesis of a bioactive peptide for the treatment of autoimmune diseases^36^. Moreover, 3-ABA has activity against different plant viruses, bacteria, nematodes and fungi^37^.

## Methods

### Molecular docking of substrates in the active site of CV_TA

The crystal structure of *Chromobacterium violaceum* (DSM30191) with PLP accommodated in the active site 4AH3^18^ was used to study the active site and select residues that can be changed to improve the activity of CV_TA towards 2-OOA and 3-OBA methyl ester. All protein preparation and modelling work was undertaken in MOE 2016.0802^38^. The docking site of the receptor was defined by selecting PLP and F88, extending by 4.5 Å. Protein Ligand Interaction Fingerprints (PLIF) analysis was undertaken on all 30 refined docking poses of each compound to determine amino acid preferred contacts.

### *In vitro* site directed mutagenesis (SDM), protein expression and purification

The transaminase gene of *C*. *violaceum* was amplified and cloned into pET-45b(+) vector as previously described^7^. Site-directed mutagenesis was performed using the QuickChange II XL Site-Directed Mutagenesis kit (Agilent Technologies, USA) according to manufacturer’s instructions. The oligonucleotide primers used for the site-directed mutagenesis are listed in Table 5. The resulting variants were verified by sequencing (GATC Biotech Hamburg, Germany). The protein expression and purification of wild type and variants was carried out as previously described^7^.

**Table 5:** Primers used for site directed mutagenesis of CV_TA.

Primer	Sequence (5′-3′)
A231S (F)	CATCCAGGGCTCCGGCGGCGTGATC
A231S (R)	GATCACGCCGCCGGAGCCCTGGATG
A231T (F)	CATCCAGGGCACCGGCGGCGTGATC
A231T (R)	GATCACGCCGCCGGTGCCCTGGATG
R416K (F)	AACAACCTGATCATGAAGGCATGCGGCGACCACATC
R416K (R)	GATGTGGTCGCCGCATGCCTTCATGATCAGGTTGTT
S156A (F)	GGCTATCACGGCGCCACCATCGGCG
S156A (R)	CGCCGATGGTGGCGCCGTGATAGCC
W60C (F)	ATGGCCGGACTGTGCTGCGTGAACGTC
W60C (R)	GACGTTCACGCAGCACAGTCCGGCCAT
Y168F (F)	GGCGGCATGAAGTTCATGCACGAGCAG
Y168F (R)	CTGCTCGTGCATGAACTTCATGCCGCC

### Activity of the wild type enzyme and SDM variants with different amino group acceptors

The activity of CV_TA towards different amino group acceptors was determined by a spectrophotometric assay based on using (*S*)-(−)-1-phenylethylamine (1-PEA) as the amino group donor and formation of acetophenone (AP) that can be measured spectrophotometrically^39^. The assay conditions were as previously described^7^.

The activity of the variants generated by SDM was tested in a 10 ml biotransformation by AP assay. The amino group donor to acceptor ratio was 4:1 (10 mM 1-PEA: 2.5 mM acceptor) except when using 3-OBA methyl ester 1:2 ratio was applied (10 mM 1-PEA: 20 mM 3-OBA methyl ester). The 10 ml biotransformation was performed using glass tubes (PYREX, England) with 0.044 mg/ml of purified enzyme at 45 °C and shaking 200 rpm. Samples (0.5 ml) were taken every 15 min for 180 min and the reaction was stopped by adding 0.2% TFA (v/v) followed by 10-fold dilution with 100 mM potassium phosphate buffer. The AP formation was then measured.

### Kinetic characterisation of CV_TA and CV_Y168F

Kinetic studies of the CV_TA activity towards the amino group donor, 1-PEA and amino group acceptors; 2-OOA and 3-OBA methyl ester; were performed by measuring the production of AP. The assay was performed under conditions previously described^7^. To study the affinity (*K*_*m*_) of CV_TA for the amino group donor 1-PEA, the concentration of 1-PEA was varied from 0.1 to 20 mM, while the acceptor, 2-OOA or 3-OBA methyl ester was fixed at 2.5 mM. The concentration of the amino group donor 1-PEA was then fixed at 10 mM and the concentration of the acceptors was varied from 0.1 to 20 mM to determine the *K*_*m*_ of CV_TA for 2-OOA and 3-OBA methyl ester.

The initial reaction rates were obtained by fitting linearly the change in absorbance (ΔAbs_245_) over time. Kinetic parameters were calculated according to the Lineweaver and Burk double reciprocal method, which allows for the determination of the Michaelis Menten constant (*K*_*m*_) and the maximum velocity of the reaction (*V*_*max*_)^40^. The results were confirmed by using non-linear regression analysis software, Enzfitter for Windows 2.0.18.0 (Elsevier, Biosoft®, UK). Using the *V*_*max*_ values the turnover number (*K*_*cat*_) and catalytic efficiency (*K*_*m*_/*K*_*ca*t_) of CV_TA was determined for each amino group acceptor^40^.

### Characterisation of the half-transamination reaction

The deamination of 1-PEA results in irreversible AP formation in the absence of an acceptor by using PLP as amino group acceptor^39^. This characteristic was used to assess the effect of mutations CV_Y168F, CV_W60C and CV_W60/Y168F on the first half of the transamination reaction and the affinity (*K*_*m*_) towards the amino donor 1-PEA. The *K*_*m*_ of variants was compared to the *K*_*m*_ of the wild type CV_TA. The reaction was set up in 1 ml total volume and it contained: 0.1–20 mM 1-PEA, 5 mM PLP, 1 mg/ml purified enzyme and 0.25% DMSO in 100 mM potassium phosphate buffer pH 7.0. It was carried out at 45 °C for 4 h with shaking at 200 rpm. Aliquots of 0.01 ml were sampled at times 0 and then every 60 min for 4 h and mixed with the same volume of 0.2% TFA (v/v) to stop the reaction, followed by 10-fold dilution with 100 mM potassium phosphate buffer pH 7.0. The formation of AP was measured spectrophotometrically at 245 nm.

## Electronic supplementary material

Supplemental Information

## Data Availability

All data and constructs are available upon request.
