# Supplementary material for: Single point mutations reveal amino acid residues important for Chromobacterium violaceum transaminase activity in the production of unnatural amino acids
Source: Sci Rep. 2018 Nov 26;8:17397. doi: 10.1038/s41598-018-35688-7 (PMC6255834; doi:10.1038/s41598-018-35688-7)
Supplement: Supplementary file 1 — Supplemental Information [file 41598_2018_35688_MOESM1_ESM.pdf]

Scientific Reports

Single point mutations reveal amino acid residues important for *Chromobacterium violaceum* transaminase activity in the production of unnatural amino acids

Sarah A. Almahboub[1], Tanja Narancic[1,2*], Darren Fayne[3], Kevin E. O'Connor[1,2]

[1]UCD Earth Institute and School of Biomolecular and Biomedical Science, University College Dublin, Belfield, Dublin 4, Ireland

[2]BEACON - Bioeconomy Research Centre, Ireland, University College Dublin, Belfield, Dublin 4, Ireland

[3]Molecular Design Group, School of Biochemistry and Immunology, Trinity Biomedical Sciences Institute, Trinity College Dublin, Dublin 2, Ireland

Running Head: Biosynthesis of unnatural amino acids

*Corresponding author: Dr Tanja Narancic School of Biomolecular and Biomedical Sciences, Earth Institute, O'Brien Centre for Science, University College Dublin, Belfield, Dublin 4, Ireland Telephone: +353 1 716 2198; Fax: +353 1 716 1183; E-mail: tanja.narancic@ucd.ie

Keywords

unnatural amino acids; ω-transaminase; *Chromobacterium violaceum* DSM30191; site-directed mutagenesis

Table S1 Reaction rate (nmoles/min/mg) of the CV_TA wild type enzyme and variants generated by SDM with different aliphatic amino acceptors and 1-PEA as amino donor.

	CV_TA							
**Amino acceptor**	**WT**	**W60C**	**S156A**	**Y168F**	**A231S**	**A231T**	**R416K**	**W60C/Y168F**
**2-OBA**	2716.2 ± 553	1828 ± 67.1	500 ± 40	2406.3 ± 124.2	1226.9 ± 3.4	166 ± 9.1	94.9 ± 18.3	1836.7 ± 27.4
**2-OBA methyl ester**	2625.6 ± 462.9	1690.1 ± 164.6	422.5 ± 22	2414.4 ± 106.7	849.3 ± 6.1	135.8 ± 15.2	60.4 ± 3	1626.5 ± 123.5
**3-OBA methyl ester**	192 ± 45.7	NA	NA	374 ± 19.8	78 ± 9.1	25 ± 5.3	39 ± 4	NA
**2-OPA**	1780.6 ± 201.9	1170.5 ± 70.1	306.1 ± 34	2166.5 ± 146.3	771.7 ± 6	79.8 ± 15.2	34.5 ± 6.1	1193.2 ± 32
**4-OPA**	129.3 ± 26.9	NA	NA	81.4 ± 22.1	NA	NA	23.7 ± 3.1	NA
**2-OHA**	4488.2 ± 347.5	2489.8 ± 118.9	538.9 ± 11	3323 ± 513.7	1304.2 ± 45.7	251.1 ± 71.6	17.3 ± 3	2953.1 ± 100.6
**3-OHA methyl ester**	NA	NA	NA	NA	NA	NA	NA	NA
**2-OOA**	778.9 ± 47.3	454.9 ± 57.9	185.4 ± 22	1122 ± 129.6	840.7 ± 85.4	129.3 ± 12.2	12.9 ± 3.1	501.2 ± 50.3

NA: No activity
OBA; oxobutyric acid, OPA; oxopentanoic acid, OHA; oxohexanoic acid and OOA: oxooctanoic acid).
All values are a mean of three independent determinations.
